# Dietary guidance on plant-based meat alternatives for individuals wanting to increase plant protein intake

**DOI:** 10.3389/fnut.2025.1641234

**Published:** 2025-08-05

**Authors:** Virginia Messina, Nanci S. Guest, Alison M. Duncan, Ann Reed Mangels, Jack Norris, Matt Ruscigno, Andrea J. Glenn, Taylor Wolfram, Christopher P. F. Marinangeli, Mark Messina

**Affiliations:** ^1^Nutrition Matters, Inc., Pittsfield, MA, United States; ^2^Department of Nutritional Science, Temerty Faculty of Medicine, University of Toronto, Toronto, ON, Canada; ^3^Department of Human Health and Nutritional Sciences, University of Guelph, Guelph, ON, Canada; ^4^Retired, Santa Cruz, CA, United States; ^5^Vegan Outreach, Sacramento, CA, United States; ^6^Consultant, Los Angeles, CA, United States; ^7^Department of Nutrition and Food Studies, New York University, New York, NY, United States; ^8^Taylor Wolfram PLLC, La Grange, IL, United States; ^9^Centre for Regulatory Research and Innovation, Protein Industries Canada, Regina, SK, Canada; ^10^Director of Nutrition Science and Research, Soy Nutrition Institute Global, Jefferson City, MO, United States

**Keywords:** environment, vegetarian, ultra-processed foods, legumes, plant-based meat

## Abstract

A new generation of plant-based meat alternatives (PBMAs) has entered the mainstream. These products contain concentrated sources of plant protein and are formulated to mimic the taste and texture of their meat-based counterparts, especially red meat. The increased availability of these products coincides with calls from health agencies to increase the dietary plant-to-animal protein ratio for health and environmental reasons. The role of PBMAs in achieving the goal of consuming more plant protein may be particularly important since consumption of whole plant foods, such as legumes, which includes pulses (e.g., beans, peas and lentils), is unlikely to increase without major public policy initiatives. Nevertheless, there is debate about the healthfulness of PBMAs and about whether the benefits associated with traditional plant-based diets emphasizing whole plant foods apply to PBMAs. These products are heavily processed, often high in sodium, and contain lower levels of compounds (e.g., fiber, resistant starch, polyphenols) typically associated with the benefits of plant-based diets. On the other hand, PBMAs are excellent sources of protein, and many are fortified with nutrients of concern in plant-based diets. Collectively, the evidence suggests that while they may not provide all the benefits of whole legumes, PBMAs have health and environmental advantages over comparable animal-derived foods. For most individuals, a daily serving of a PBMA fits well within the context of an overall healthy diet. Higher intakes may also be compatible with healthy eating, especially for those whose protein and/or calorie needs are increased.

## Introduction

Over the past decades, several protein-rich plant foods such as tofu, tempeh, seitan, and textured vegetable (soy) protein (TVP) have made inroads among mainstream consumers. However, none of these have achieved the same level of adoption as the newest generation of plant-based meat alternatives (PBMAs) (1). These modern alternatives closely mimic the organoleptic characteristics and nutritional profile of their meat-based counterparts through enhanced orosensory properties and nutrient fortification (2). And unlike previous meat alternatives that were mainly available in niche health food stores, PBMAs are now widely available in grocery chains and restaurants. Researchers in countries including Hong Kong (3), South Africa (4), Germany (5–7), Italy (6, 8), Belgium (9), United Kingdom (6, 10–12), Singapore (13), France (6), Spain (6, 14–16), New Zealand (17), Canada (11, 18), Australia (19), Greece (20), United States (11, 21, 22), the Netherlands (23), Malaysia (24), and Sweden (25) have recognized this growing trend and have conducted extensive nutritional analyses of PBMAs to better understand their potential contribution to meeting nutrient needs in various dietary patterns.

Plant foods aimed at replacing meat have existed for centuries. For example, tofu has been consumed for two millennia (26). Meat alternatives made from peanuts and grains were available in the late 19th century in the United States and were developed specifically to improve health (27, 28). The 1960s saw the emergence of “veggie burgers” made primarily from legumes, such as dried beans, which targeted both vegetarian and nonvegetarian health-conscious consumers (29). A significant advancement came in the form of TVP, which is produced by heating defatted soy meal through extrusion texturization, altering its physical and chemical properties and creating a meat-like texture. TVP was seen as a sustainable product and versatile ingredient in early developments of meat substitutes (30). The evolving formulations of PBMAs warrant a close examination of their health effects and rationale for their inclusion in the diet. Although there are also mycoprotein-based meat alternatives (31), the discussion that follows specifically focuses on alternatives to meat that are based on proteins derived from plants. The intent of this perspective is to provide health professionals with brief background information on PBMAs designed specifically to replace red meat (beef), to highlight nutritional considerations relevant to incorporating PBMAs into the diet, and to offer guidance on intake recommendations.

## Rationale for PBMAs: increasing the dietary plant-to-animal protein ratio

The emergence of PBMAs aligns with increasing calls, for both environmental (32, 33) and health (34–36) reasons, for high-income nations to adopt more sustainable plant-forward diets through the replacement of some animal protein with plant-based options. PBMAs generally consist of a concentrated source of one or more proteins, often extracts from legumes such as soybeans or peas, but also wheat protein, usually classified as an isolate (≥90% protein) or concentrate (≥65% protein) (37), along with fat, binding and flavoring agents, and colorants. While the protein content of PBMAs can vary, products mimicking beef burgers typically have 15–20 g of protein per 100 g serving (Table 1) (10, 12, 18, 38–40).

**Table 1:** Nutrient content of beef and widely available plant-based (vegan) meat alternatives in North America and Europe.

Nutrient composition	Impossible burger	Beyond burger	Gardein ultimate burger	MorningStar original griller	Boca original vegan	Emerge plantbased burger	Dr. Praeger’s perfect burger	Heura burger	Meatless farm burger	Ground beef (80% lean, 20% fat)
Serving size (g)	113	NI^*^	113	64	71	113	113	100	100	100
Energy (kcal)	230	230	210	140	80	220	220	207	217	248
Protein (g)	19	21	20	16	14	20	20	19	16.8	17.5
Protein (% kcal)	33.0	36.5	38.1	45.7	70.0	34.8	36.4	36.7	31	28.2
Total fat (g)	13	14	14.0	6	1	14	12	12	14.2	19.4
Total fat (% kcal)	50.9	54.8	60	38.6	11.3	220	49.1	52.2	59	70.4
Saturated fat (g)	6	2	6	1	0	9	1	3	2.9	6.84
Polyunsaturated fat (g)	NI	2.5	6	NI	NI	NI	NI	NI	NI	0.485
Monounsaturated fat (g)	NI	8.0	NI	NI	NI	NI	NI	NI	NI	7.25
Trans fat (g)	0	0	0	0	0	0	0	NI	NI	0.7
Cholesterol (mg)	0	0	0	0	0	0	0	NI	NI	68
Total carbohydrate (g)	9	8	5	8	7	6	9	4.2	4.1	0
Fiber (g)	5	2	2	3	4	0	3	3.1	4.5	0
Total sugars (g)	<1	0	1	2	0	0	<1	0.7	1.2	0
Added sugars (g)	<1	0	<1	<1	0	0	0	NI	NI	0
Sodium (mg)	370	310	360	320	440	390	410	380	481	55
Vitamin D (ug)	0	0	0	0	0	0	0	NI	NI	NI
Calcium (mg)	180	120	0	50	80	41	70	NI	NI	7
Iron (mg)	4.2	4	3.5	1.1	2.4	6	5.8	10	NI	1.96
Potassium (mg)	700	370	150	230	420	136	100	NI	NI	273
Phosphorus (mg)	190	NI	NI	NI	NI	NI	NI	NI	NI	144
Zinc (mg)	5.50	NI	NI	NI	NI	NI	NI	NI	NI	3.85

^*^NI, not indicated. Sources: Ground beef (FoodData Central, FDC ID: 2514744 NDB Number:23572: https://fdc.nal.usda.gov/food-details/2514744/nutrients). Impossible: https://impossiblefoods.com/beef/plant-based-impossible-burger. The Impossible burger also contains 0.46 mg thiamin, 0.19 mg riboflavin, 9.30 mg niacin, 0.34 mg vitamin B6, 85.0 ug folate and 3.01 ug vitamin B12. Beyond: https://www.beyondmeat.com/en-US/products/the-beyond-burger. Gardein: https://www.gardein.com/beefless-and-porkless/classics/ultimate-plant-based-burger. MorningStar: https://smartlabel.kelloggs.com/Product/Index/00028989100801. Boca: https://www.kraftheinz.com/boca/products/00759283334455-original-vegan-veggie-burgers. Preager’s perfect burger: https://www.drpraegers.com/products/plant-based-perfect-burger. Heura Burger: https://heurafoods.com/products/burgers-plant-based-protein. Also contains 2 ug vitamin B12. Emerge: https://www.nutritionix.com/i/simple-truth/emerge-plant-based-patties/5e2bebefded6acd81b42820a. Meatless Farm: https://meatlessfarm.com/our-products/fresh-plant-based-burgers/.

High-income countries currently derive roughly two-thirds of their dietary protein from animal sources and one-third from plants (41, 42). Although efforts such as the EAT-Lancet Commission on Food, Planet, Health have called for a dramatic reduction in animal protein intake (32), a more practical and achievable goal for the near future may be to balance protein intake equally between plant and animal sources. Epidemiologic evidence suggests this approach is likely to result in health benefits. A recent analysis of the Nurses’ Health Studies I & II and Health Professionals Follow-up Study, found that a plant-to-animal protein of roughly 1:1.3 was associated with a 27% lower risk of coronary heart disease compared to a ratio of approximately 1:4.2, with a likelihood of further protection with even higher intakes of plant protein and corresponding reductions in animal protein intake (43).

Previous estimates have indicated that consuming PBMAs four times per week in place of meat would reduce the ratio of plant to animal protein in the diets of high-income countries from about 1: 2 to almost 1:1 (44). Because PBMAs may not provide all the benefits of traditionally prepared legumes, guidance on varying sources of plant protein is warranted. The fiber, resistant starch (45), and bioactives (46) in whole legumes may act additively and synergistically to produce health benefits (47, 48). These components can be decreased in food products that use extracted protein sources. Based on nutrient composition and limited clinical research, evidence suggests that a daily serving of a PBMA fits well within the context of an overall healthy diet. Whether higher intakes are also compatible with healthy eating, especially for those whose protein and/or calorie needs are increased, will depend on the overall content of the diet. The key findings supporting the incorporation of PBMAs into the diet are highlighted in Box 1.

BOX 1 **Key points related to the incorporation of PBMAs into the diet.**

1. The new generation of PBMAs provide a convenient means by which consumers in high income countries can increase their dietary plant-to-animal protein ratio.

2. Unlike earlier meat substitutes, which often had distinct tastes or textures, newer PBMAs closely replicate the experience of eating meat.

3. Debate exists about the extent to which the health benefits associated with plant-based diets apply to PBMAs.

4. PBMAs can contain fiber and many are lower in saturated fat while providing similar amounts of protein as meat, but are often high in sodium and may not contain, or contain in lower amounts, some beneficial plant compounds.

5. The composition of PBMAs widely varies depending upon primary sources of fat and protein and the degree of nutrient fortification.

6. PBMAs are versatile and can fit into traditional dietary habits and patterns.

## PBMAs provide a means for increasing plant protein intake

Increased intake of dried beans, nuts, seeds and soy products is one approach toward increasing the dietary plant-to-animal protein ratio. Legumes are rich in protein and fiber (49, 50), affordable (51), have a small environmental footprint (52, 53), and have several reported health benefits (54–56). In particular, legume-rich diets are associated with a more optimum nutrient intake (57).

However, despite evidence that legume consumption improves the nutrient density of the diet (58), these foods are a relatively minor protein source in high-income countries (59–61). Indeed, even among vegans, legume intake is relatively low (50, 62, 63). Numerous real and perceived barriers to consuming legumes such as dried beans cooked in the traditional manner (boiled) are well-documented (64). These barriers include the time and effort required for cooking, possible gastrointestinal disturbances, dislike of the taste and texture and the association of legumes with lower socioeconomic status (poor man’s meat). There is little reason to think that consumption will substantially increase without greater public policy initiatives (64–66).

## Health effects of PBMAs

### PBMAs are Nova-classified as ultra-processed foods

Given their convenience, improved orosensory properties (67), and similar culinary role to the products they are intended to replace, it is reasonable to expect that modern PBMAs will continue their growth in popularity among consumers and will be seen as acceptable options by nutrition professionals. Despite these positive attributes, there are concerns about the lack of data on the long-term health effects of regular consumption of these products (68) and some have cautioned against assuming that the benefits associated with consuming plant protein from whole foods applies to PBMAs (2, 69, 70).

The Nova food classification system classifies the new generation of PBMAs as ultra-processed foods (UPFs) because of their formulation, which can include additives such as emulsifiers, and because of the processing involved in the ingredients they contain, such as concentrated sources of protein (71). Proponents of Nova recommend avoiding UPFs as much as possible (72, 73), citing the many observational studies linking UPF intake with a wide range of adverse health outcomes (74–78). Recent research indicates that consumers view PBMAs more unfavorably than other categories of equally processed foods (79). In fact, a recently published survey of French consumers found more respondents consider UPFs to be a cancer risk factor than red and processed meat (80). While research is needed to better understand how the processing and formulation of foods may affect health, several lines of evidence suggest that Nova is insufficiently nuanced to serve as a consumer guide for food purchasing decisions.

Many UPFs are highly rated by nutrient profiling models (81–83) and have demonstrated health benefits (84, 85). For example, a recent systematic review and meta-analysis that included 17 randomized controlled trials (RCTs) compared the effects on health outcomes of cow’s milk, a Nova group 1 food (unprocessed/minimally processed) to soymilk, a Nova group 4 food (ultra-processed). Results showed that in comparison to cow’s milk, soymilk lowered blood pressure, low-density lipoprotein cholesterol (LDL-C) levels, and inflammation (84).

As noted, total UPF intake has been linked with a range of adverse health outcomes (74–78), including type 2 diabetes (T2D), cardiovascular disease (CVD) and cancer (85). However, although many subcategories of UPFs, such as processed meats and sweetened beverages, have been linked with harmful effects, several reports have found that other UPFs, including PBMAs, breads and cereals, are not linked with harmful effects or are associated with improved health status (86–88). Particularly relevant are the findings that plant-based UPFs and animal-based UPFs often exhibit opposite effects. For example, plant-based UPFs were linked with a decreased risk of developing T2D in a recent prospective analysis of the European Investigation into Cancer and Nutrition (EPIC) cohort (88), and with longer leukocyte telomere length (LTL) in a cross-sectional analysis of the UK Biobank (89) whereas intake of animal-based UPFs was associated with an increased risk of T2D (88) and shorter LTL (89). Also of potential relevance is the conclusion from one analysis that none of the common attributes of UPFs (e.g., high caloric density, fast eating rate, fast energy intake rate, soft texture, hyper-palatable, inexpensive, and low satiety) apply to PBMAs more so than to beef, a food designated as unprocessed/minimally processed (group 1) (38).

Finally, one recently published analysis of data from the UK Biobank that included over 126,000 adults and a median follow-up of 9 years found that intake of plant-sourced UPFs was associated with an increased risk of CVD and total mortality (90). However, because the meat alternative category represented only 0.2% of daily energy intake (~4 kcal), the intake levels of these foods were not large enough to provide meaningful insight about their health impacts. Furthermore, this food group included several products such as tofu and tempeh, suggesting that intake of PBMAs was negligible. Also, in the Cardiovascular Health Study, both plant and animal-sourced UPFs were associated with an increased risk of all-cause mortality. This study is a prospective analysis of 2,582 participants (median age: 77 years) who were followed up for 10 years during which time there were 2,242 deaths. However, the plant-sourced UPF category did not include PBMAs (or plant-based dairy alternatives) but instead was populated by foods such as candy bars, brownies, potato chips and crackers (122).

### Randomized controlled trials involving PBMAs

Although limited clinical research involving PBMAs has been conducted, some evidence shows that PBMAs lead to improved markers of cardiometabolic risk compared to meat (91–93). For example, one RCT showed reductions in body weight and lower circulating levels of LDL-C and trimethylamine-N-oxide (TMAO) when participants consumed approximately 2.5 servings daily of PBMAs versus an equivalent amount of meat products (91). This trial also found that the PBMAs led to lower urinary excretion of sulfate, ammonium, phosphorus, and nitrogen and higher urinary excretion of citrate, suggesting benefits for patients with kidney disease (94). No differences between diets were observed for other outcomes such as blood pressure and inflammation (91, 95). However, a comparable trial measuring similar outcomes showed no differences in LDL-C, body weight, or TMAO (92), most likely due to the differences in the nutrient composition of the PBMAs used in these two comparable trials. These findings suggest that the focus should be on nutrient content, not on the degree of processing.

Although long-term data on the health impact of the new generation of PBMAs is unavailable, over the past several decades, a considerable amount of clinical work has examined the health effects of a range of concentrated sources of protein including soy (96), wheat (97), and to a lesser extent, pea protein (98). There is also evidence upon which to evaluate the healthfulness of the various sources of fat used in PBMAs (99, 100). Thus, considerable information on the two main ingredients in PBMAs is readily available. Therefore, although the new generation of PBMAs are novel, from a nutritional perspective, their likely health impact can be surmised from the existing research on their main ingredients and the clinical work directly involving these products conducted thus far.

### Potential indirect benefits of incorporating PBMAs into the diet

By aiding in the reduction of red meat intake, PBMAs may indirectly contribute to improved health outcomes since red meat is linked with an increased risk of several chronic diseases, including T2D (101), CVD (102), and various cancers (103, 104). Depending upon the specific product, PBMAs may help increase fiber and decrease saturated fat intake if they are replacing animal products. There is also likely an environmental benefit since even with the processing involved in the production of PBMAs, life-cycle analyses indicate these foods have a lower impact than their meat-based counterparts (10, 105).

In general, the digestibility and quality of plant protein is lower than animal protein (106–108). However, protein is not a nutrient of concern for vegetarians or omnivores in high-income countries. Furthermore, differences between plant and animal proteins noted in acute studies in which muscle protein synthesis is assessed, or based on digestibility and amino acid composition, are not apparent in longer-term studies measuring muscle protein synthesis (109, 110) and gains in muscle mass and strength (111). While the quality of the protein in PBMAs will vary according to the source, pea protein and soy protein, which are the main sources of protein currently found in PMBAs, are well-digested and provide good amounts of essential amino acids (106, 107, 112, 113). Given that many PBMAs provide equivalent amounts of protein per serving as red meat, and twice that of dried beans (50), protein nutriture is unlikely to be compromised by the inclusion of PBMAs in the diet.

### Potential to enhance the nutrient content of PBMAs

PBMAs can be fortified with nutrients such as iron, zinc, selenium, iodine, and vitamin B12 to increase their nutritional similarity to corresponding animal products. The addition of vitamin B12 to these products would be of particular benefit to those consuming plant-based diets, but among the PMBAs listed in Table 1, only one is fortified with this nutrient. Very limited information is available on the bioavailability of added nutrients in PBMAs, although considerable data are available on the absorption of iron from soybeans and from various concentrated sources of soy protein (114). Although the absorption of nonheme iron from plants is lower than the absorption of heme iron from animal products, evidence that vegan diets increase risk of anemia is unimpressive (123). Although speculative, recent evidence suggests vegans absorb iron more efficiently than omnivores (117). PBMAs are likely to be relatively low in inhibitors of mineral absorption, such as fiber, phytate, and polyphenols, but until otherwise shown, it is reasonable to assume that many of these fortificants will be less well-absorbed from PBMAs compared to meat (50, 115, 116). Whether mineral bioavailability is better than or comparable to that of dried beans is unclear, but the potential for nutrient fortification is likely to be an advantage of PBMAs. For example, the iron content per serving of PBMAs is roughly twice that of dried beans (~2.3 mg/100 g cooked) (114) and lean ground beef (Table 1) (117).

The adequate intake for zinc established by the European Food Safety Authority is based on the phytate content of the diet (118), which typically increases as the diet contains more plant foods. Manufacturers of PBMAs would be well-advised to consider fortifying their products with zinc. Only one of the PBMAs included in Table 1 is fortified with this mineral (5.5 mg/serving).

Sodium content can be high in PBMAs (44, 119), although these foods may contain more or less sodium than comparable meat products, depending on how much salt is added during meat preparation (12, 13, 120). For individuals on low-sodium diets (≤1,500 mg/d) (121), most PBMAs could not be consumed on a daily basis. However, the nutrient composition of PBMAs varies markedly and consumers who limit dietary sodium can be guided toward lower-sodium products. Furthermore, there are often reformulations aimed at improving nutrient content. For example, Impossible Foods now has a “lite” version of their burger which is much lower in saturated fat and Beyond Meat recently switched from using coconut oil to avocado oil in their burger, which markedly reduced its saturated fat content. Although some PBMAs may still be relatively high in saturated fat, the amounts are not higher than what would be found in lean ground beef. By working closely with consumers, dietitians can guide the selection of PBMAs that align with individual needs, while ensuring that these foods contribute meaningfully to their diet and promote overall healthy eating patterns. It is important for consumers to understand that there is a large variability in the nutrient content of PBMAs and for this reason, all PBMAs should not be similarly viewed from a health and nutritional perspective.

## Discussion

As discussed, in recent years several criticisms about the role of PBMAs in a healthy diet have been raised. Notable among them is that these products are Nova-classified as UPFs. However, although in most instances UPFs are designed to displace more healthful foods in the diet, such as chicken nuggets for the replacement of chicken, an argument can be made that in the case of PBMAs, they are displacing a less healthful food, as red meat is linked with an increased risk of several chronic diseases. Collectively, evidence indicates that PBMAs are a healthful option for increasing plant protein intake. They may also serve as a gateway to more plant-focused diets, which may result in greater consumption of whole plant foods. From a practical perspective, the replacement of meat with a PBMA may be easier and cause less cognitive dissonance than replacing meat with tofu, beans, or lentils. Because PBMAs can be fortified, there is the potential for these products to more closely match the nutrient content of the foods they are intended to replace compared to dried beans consumed in the traditional manner.

Increasingly, PBMAs are incorporating a variety of legumes and ingredient derivatives that provide consumers with nutrient-dense products with a favorable sensory experience. Therefore, PBMAs can potentially aid in the transition to the long-term adoption of more plant-based dietary patterns. Nevertheless, the popularity of PBMAs suggests the need for research aimed at better understanding their health effects. It is important that dietary intake instruments accurately assess the intake of different types of PBMAs so more insight from observational studies can be gained and for longer-term RCTs involving PBMAs to be conducted.

## Data Availability

The original contributions presented in the study are included in the article/supplementary material, further inquiries can be directed to the corresponding author/s.
